# Growth Attenuation with Developmental Schedule Progression in Embryos and Early Larvae of *Sterechinus neumayeri* Raised under Elevated CO_2_

**DOI:** 10.1371/journal.pone.0052448

**Published:** 2013-01-02

**Authors:** Pauline C. Yu, Mary A. Sewell, Paul G. Matson, Emily B. Rivest, Lydia Kapsenberg, Gretchen E. Hofmann

**Affiliations:** 1 Department of Ecology Evolution and Marine Biology, University of California Santa Barbara, Santa Barbara, California, United States of America; 2 School of Biological Sciences, University of Auckland, Auckland, New Zealand; Institute of Marine Research, Norway

## Abstract

The Southern Ocean, a region that will be an ocean acidification hotspot in the near future, is home to a uniquely adapted fauna that includes a diversity of lightly-calcified invertebrates. We exposed the larvae of the echinoid *Sterechinus neumayeri* to environmental levels of CO_2_ in McMurdo Sound (control: 410 µatm, Ω = 1.35) and mildly elevated pCO_2_ levels, both near the level of the aragonite saturation horizon (510 µatm pCO_2_, Ω = 1.12), and to under-saturating conditions (730 µatm, Ω = 0.82). Early embryological development was normal under these conditions with the exception of the hatching process, which was slightly delayed. Appearance of the initial calcium carbonate (CaCO_3_) spicule nuclei among the primary mesenchyme cells of the gastrulae was synchronous between control and elevated pCO_2_ treatments. However, by prism (7 days after the initial appearance of the spicule nucleus), elongating arm rod spicules were already significantly shorter in the highest CO_2_ treatment. Unfed larvae in the 730 µatm pCO_2_ treatment remained significantly smaller than unfed control larvae at days 15–30, and larvae in the 510 µatm treatment were significantly smaller at day 20. At day 30, the arm lengths were more differentiated between 730 µatm and control CO_2_ treatments than were body lengths as components of total length. Arm length is the most plastic morphological aspect of the echinopluteus, and appears to exhibit the greatest response to high pCO_2_/low pH/low carbonate, even in the absence of food. Thus, while the effects of elevated pCO_2_ representative of near future climate scenarios are proportionally minor on these early developmental stages, the longer term effects on these long-lived invertebrates is still unknown.

## Introduction

The Southern Ocean is predicted to be one of the first major regions to experience the biological consequences of ocean acidification [1], [2]. Areas of the global oceans predicted to rapidly become low to undersaturated (Ω <1) with respect to calcium carbonate (CaCO_3_) are considered hotspots of ocean acidification; the waters surrounding Antarctica are particularly close to undersaturation due to both the interaction of seawater with CO_2_ at cold temperatures and the transport of remineralized deep water from the conveyor belt [1], [3], [4]. These changes to ocean chemistry render the calcified biota of the Southern Ocean especially vulnerable compared to warmer, lower latitude coastal areas, because Antarctic organisms already inhabit waters where forming calcium carbonate is more challenging [5], [6], [7] and are under threat from incursions by decapod predators [8], [9], [10]. Presently there is emerging data regarding the response of various Antarctic invertebrates to altered seawater carbonate chemistry [11], [12], [13], [14]. To explore the impacts of future acidification due to anthropogenic CO_2_ inputs on developmental stability in a key benthic echinoderm, we utilized the sea urchin *Sterechinus neumayeri* to test the effects of high pCO_2_/low pH on early development and larval growth.

We selected an echinoid for our study as echinoderms in general are an important macrofaunal contributor to the carbonate geochemistry of global oceans, and *S. neumayeri* is one of the main contributing species to the standing stock of carbonate in well-sampled regions of Antarctica [15]. They are (along with the asteroid *Odontaster validus*) among the most abundant calcifying benthic species in the shallow benthos and are a dominant grazer species [16]. Because of their relative importance, the effects of climate change, and in particular, ocean acidification, on these organisms is a topic of concern [3], [6].

Interestingly, despite the vulnerability of the Antarctic marine ecosystem, we have limited information about present day carbonate chemistry in the Southern Ocean and coastal regions of particular biological importance, such as the Western Antarctic Peninsula. The carbonate chemistry of the Southern Ocean has generally been assessed during oceanographic cruises (e.g. WOCE, JGOFS: [4], [17], and others: [18], [19]). Recent efforts to enhance and coordinate oceanographic monitoring in the Southern Ocean have been initiated (Southern Ocean Observing System (SOOS) – www.soos.aq). However, nearshore measurements in the vicinity of shallow benthic macrofaunal assemblages are rare [20]. The availability of new equipment – autonomous pH sensors based on ion-sensitive field-effect transistor (ISFET) pH electrodes - to measure the environmental pH [21] has allowed us to use these data [20] to set the control conditions for our laboratory experiments. These new high frequency time-series measurements indicate that coastal waters near Ross Island (at depths and sites where *S. neumayeri* are present) were pH 8.02–8.04 on average during the austral spring of 2010 [20]. The pH profiles indicate that indeed these subzero waters are somewhat more acidic than warmer open ocean waters of the northeastern Pacific and other global ocean time-series locations (e.g. Hawaii Ocean Time Series), and also that the variability around Ross Island as compared to other coastal regions is low [22]. Under near-future IPCC models of the “business as usual” A1FI scenario (2007), the surface waters of the Southern Ocean will be undersaturated on average in less than 50 years time; we chose to bracket our experimentally elevated pCO_2_ levels slightly below and above that estimate to determine physiological responses to those challenges. Additionally, the oceanography of the Southern Ocean is expected to accelerate the undersaturation of shallow waters due to interactions with carbonate-poor deep water during the austral winter [4]; depending on the developmental schedule and spawn timing of *S. neumayeri*, these winter months of low calcium carbonate saturation could coincide with larval settlement [23].

In light of the unique biology of polar organisms and the accelerated pace of changes in these regions, the adaptability of polar organisms to future environmental conditions has become a research focus for the polar biology community, with a particular emphasis on adaptive physiology [22], [24], [25]. Recent studies examining the effects of ocean acidification on polar invertebrates have found mostly negative effects on calcifying species [11], [12], [14], [26], [27], [28], but a few studies found no effects on fertilization or early development in Antarctic urchins and non-calcifying species [13], [29], [30]. The breadth of the study organisms is obviously limited, and several studies were conducted using temperate seawater sources. Studies on adult organisms have also predominated due to availability of baseline physiology data on particular species, while in contrast the developmental physiology of *S. neumayeri* is well-characterized from prior research [31], [32], [33], [34], thus making it an excellent system for ocean acidification studies on early life history stages.

A growing body of literature suggests that early life history stages may be vulnerable to ocean acidification [35], [36] (see review in [37]). Given the low carbonate saturation conditions, and the long pelagic larval duration of planktotrophic Antarctic echinoderms [23], investigation of the tolerance of these species to elevated pCO_2_ includes an extended temporal dimension not possible with some temperate or tropical species: development to pluteus in *S. neumayeri* occurs in ∼17 days at −0.3°C compared to 5 days to pluteus at 17°C in the temperate confamilial species *Echinus esculentus* [38]. Thus, considering the variation of physiological responses to ocean acidification that has been observed between species of echinoids [37], [39], *S. neumayeri* provides an important study model for this environment. The slow developmental rates of *S. neumayeri* provide ample opportunity to look for morphological signs of developmental delay, especially during critical developmental events such as gastrulation, and early skeleton deposition. For this study we employed a culturing apparatus (described in [40]), which allows us to raise embryos through advanced 4-arm pluteus at different pCO_2_s in continuous flow-through cultures. Our results provide a mixed picture for this Antarctic species where no early developmental delay at benchmark morphological development points was observed under elevated pCO_2_, but significant morphometric differences between treatments were observed later in development. Allometric variation was altered under elevated pCO_2_, suggesting that growth differences may contribute to observed size differences. The negative growth response of these larvae to pCO_2_ levels of near-future climate scenarios suggests that negative impacts on calcifying species will manifest sooner in hotspots of ocean acidification such as the Antarctic.

## Methods

### Animal Collection and Culturing

Adult *Sterechinus neumayeri* were collected by SCUBA from a benthic site in McMurdo Sound near Cape Evans, Ross Island, Antarctica (S 77 degrees 38.059′ E 166 degrees 24.905′). No permits or permissions were required for the collection of animals at this location and the study site is not privately owned. *S neumayeri* is not an endangered or protected species, and adult animals were returned to the collection site at the end of the experiment. Adult urchins were maintained in seawater tables in the aquarium at the A. P. Crary Science and Engineering Center (hereafter referred to as “Crary Lab”) at McMurdo Station, Antarctica, at −1.5 to −1.0°C (ambient incoming seawater temperature) until spawning. Spawning was induced by intracoelomic injection of ice-cold 0.5M KCl. Eggs from 20 females were collected into 0.35 µm-filtered seawater and kept on ice, while sperm was collected “dry” from a single male and kept on ice until use. Eggs were combined, and fertilized together as a single batch and enumerated. Embryos were checked for fertilization success (>95%) and distributed equally into 15 buckets (5 replicate buckets for each pCO_2_ treatment level, see next section) at 10 embryos ml^−1^ within 1 hr of fertilization.

Embryos and larvae were cultured in the CO_2_ culturing system described in Fangue *et al.* [40] for 30 days. At intervals, larvae were sampled by reverse siphoning and combined between replicate buckets for analysis. Samples were fixed in 4% formaldehyde, saturating NaBO_3_-buffered seawater (pH = 9.0) and stored at 4°C for developmental stage scoring. Samples for microscopy and morphometrics were kept live on ice until ready for imaging the same day of collection; this was routinely conducted within 18 hours of sampling.

While larvae of *S. neumayeri* are competent to feed at the “prism” stage (roughly 2 weeks post-fertilization in our cultures) we chose not to feed the larvae in this experiment, for both logistical and experimental reasons. First and foremost, we do not have single-species cultures of Ross Sea phytoplankton optimized for feeding echinoderm larvae. Secondly, previous studies with fed larvae of *S. neumayeri* utilized non-native temperate phytoplankton [23], [34]; this approach is unfeasible with the flow-through system [40] as it presents an unacceptable biosecurity risk. While we recognize that maintaining pluteus larvae unfed for 2 weeks might impose an additional physiological stressor, Marsh *et al.* [34] demonstrated that total larval lengths ([34] Fig. 3A: “body”) of unfed larvae (57d) were not statistically different from larvae cultured with “natural” seawater (filtered to 80 µm, but without algal amendment) during the late austral spring in McMurdo Sound, when there are low food conditions [41]. Further, arm lengths of fed and unfed larvae were not statistically different until 40 days of age [34]; this invariance is within the time frame of the current experiments, and therefore the absence of food would not necessarily affect our results.

### CO_2_ Culturing System

Three treatment pCO_2_ levels (410-control, 510 and 730 µatm) and 5 replicates per treatment were established. Modifications to the system [40] included the use of additional insulation on water tubing to maintain temperatures near −1.5°C, and pumping gas at the treatment levels into the headspace of the buckets. Seawater chemistry was monitored via daily pH readings (spectrophotometric pH_TS_: [41]) (Shimadzu Instruments, Japan) and Total Alkalinity measurements (open-cell titration: [41]) (Mettler-Toledo). Temperature and pH of all buckets, both reservoirs and larval culture buckets, were monitored. Daily Total Alkalinity samples from treatment reservoirs were poisoned with mercuric chloride (0.02%: [42]) for preservation during storage prior to analysis. Salinity was measured from discrete reservoir samples (3100 Conductivity Meter, YSI). Carbonate chemistry parameters were calculated using CO2Calc [43].

### Developmental Stage Scoring

Embryo/larval samples were combined from the five replicate culture buckets for morphological staging to average out any minor temperature effects and to smooth out differences between replicate cultures as samples for morphometrics were also pooled [44]. Samples were staged on a Sedgewick Rafter slide and transects run across the slide until at least 200 embryos/larvae were staged. Development was scored at 10 developmental points during development, at intervals corresponding to major developmental and morphological changes [23], [34].

### Microscopy and Image Analysis

Larvae were gently fixed with a 10 ul drop of 4% NaBO_3_ buffered formaldehyde and immediately wet-mounted on slides for photography. Brightfield and Phase images were captured with a Zeiss Axioskop 50 and a SpotCAM. Brightfield DIC and Polarized light images were captured with a Nikon Petrographic Scope and a Canon PowershotA630. All images were calibrated with a stage reticule with 10 µm resolution and analyzed with ImageJ.

**Figure 1:**
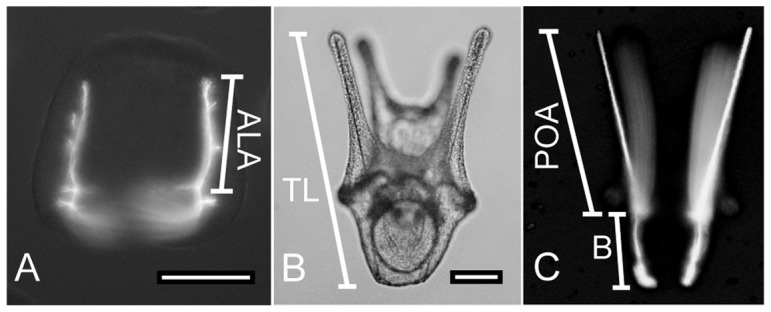
Morphometric measurements of spicule elements and total lengths at prism and pluteus stages. Scale bar shown in lower right corner of A and B = 100 µm. A) A prism stage larva (15 days) visualized under cross-polarized illumination. The ALA rod length (ALA) was measured from the origin of the triradiate center to the tip of the spicule rod. B and C) A pluteus larva (30 days) visualized under brightfield (B) and cross-polarized illumination (C). Plutei oriented with their oral side facing up were imaged with the postoral arms in the same plane of focus as the aboral tip of the larva. Total length (TL) was measured from the tip of the postoral arm rod to the aboral tip of the body. Postoral arm rod length (POA) was measured from the tip of the postoral arm rod to the origin of the triradiate center, and body rod spicule length (B) was measured from the origin of the triradiate center to the aboral tip of the body rod.

Morphometric measurements of spicules and total lengths are shown in Fig. 1. At days 12 and 15, the nascent anterolateral arm (ALA) rod of late gastrulae and prisms was the axis most easily visualized with the embryos lying on the ventral surface. The ALA rod length was measured from the origin of the triradiate center to the tip of the rod (Fig. 1A). At days 20 and 30, only plutei oriented with their oral side facing up were imaged, so as to keep the postoral arms in the same plane of focus as the aboral tip of the larva (Fig. 1B). Postoral arm (POA) rod length and body rod length (Fig. 1C) were measured independently of the total body length on a polarized light duplicate image.

### Statistical Analyses

Regressions, ANOVAs and F-tests were performed using MS Excel with the StatPlus:mac plug-in (AnalystSoft Inc.). Due to the high variance in morphometric measurements between individuals, PERMANOVA [45], which is insensitive to non-normality of data, was employed for statistical analyses. Permutational analysis for homogeneity of multivariate dispersion, PERMANOVA and permutational pairwise analyses were performed on untransformed morphometric data using the “vegan” package [46] for R, and the independent PERMANOVA and PERMDISP2 programs [47].

## Results

### Culturing Conditions

**Figure 2:**
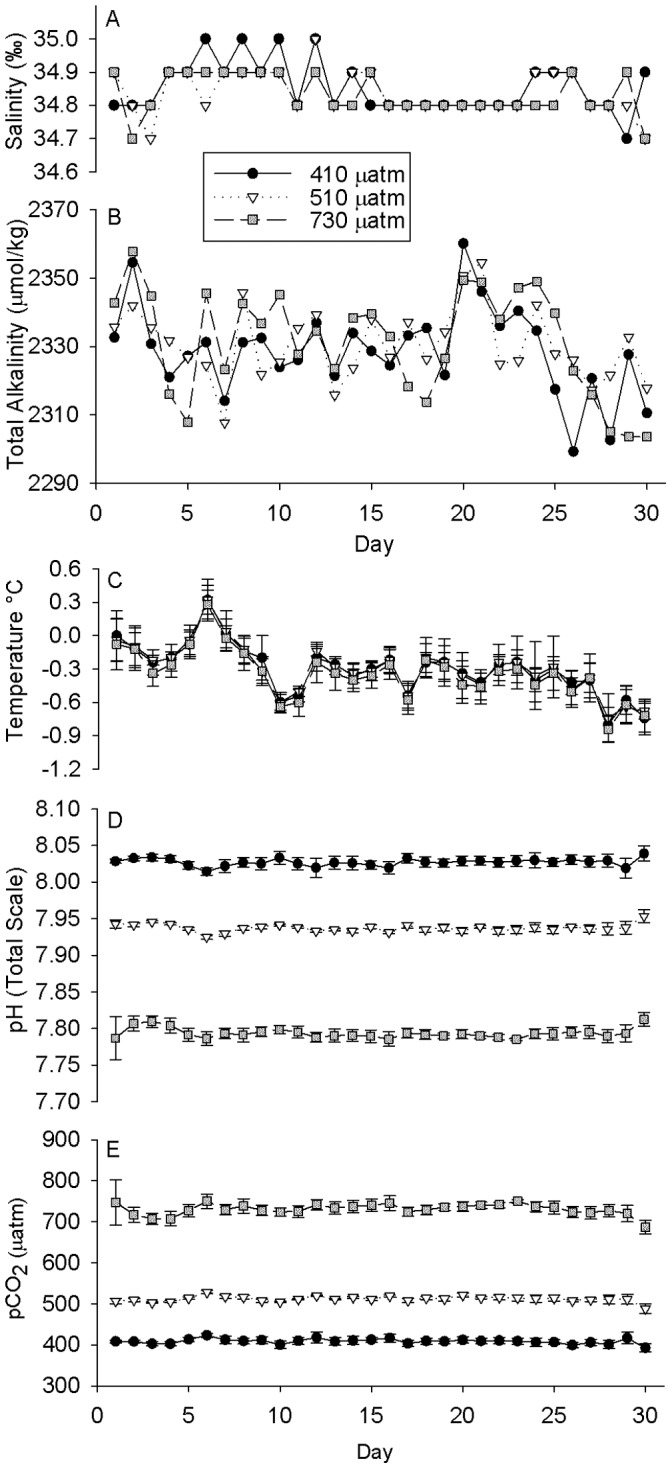
Summary of seawater chemistry. Daily measurements of salinity (A) and Total Alkalinity (B) in the three reservoir buckets for the three treatment levels 410, 510 and 730 µatm. Total Alkalinity was not significantly different between treatments over the duration of the experiment (n = 30, ANOVA, p>0.1). Averaged daily measurements of temperature (C) and pH_TS_ (D) in 5 replicate culture vessels per treatment (± SD). Calculated average pCO_2_ (E) of 5 replicate culture vessels per treatment (± SD).

Once stable carbonate chemistry conditions were established in larval culture containers, we were able to culture embryos continuously for 30 days, from shortly after fertilization to advanced 4-armed pluteus. Seawater conditions in the Crary Lab aquarium were fairly representative of water conditions in McMurdo Sound. The salinity was almost invariant (Fig. 2A), remaining at 34.8–34.9‰ over the course of the 30-day experiment. The alkalinity values (Fig. 2B) were slightly more variable, but were not significantly different between treatments over the time-course (n = 30, ANOVA, p>0.1) with the average alkalinities of the three reservoirs running at 2328.5 (±13.0 SD), 2330.5 (±10.5) and 2331.3 (±15.5) for the 410 µatm (control), 510 µatm and 730 µatm pCO_2_ treatments respectively. While the environmental seawater temperature was −1.9°C in McMurdo Sound, the incoming seawater in Crary Lab was consistently over a degree warmer, especially given the controlled air temperature within the lab. Water temperatures (Fig. 2C) averaged −0.3 (±0.2), −0.3 (±0.2) and −0.4 (±0.2) °C (n = 5 each treatment, ± SD) for the three treatment levels (410 control, 510 and 730 µatm respectively), with the overall average over time being −0.3°C (±0.2 SD). Averaged pH_TS_ (Fig. 2D) and pCO_2_ (Fig. 2E) (n = 5 each treatment, ± SD) over the time course of the experiment showed acceptable consistency within each treatment despite the variation in alkalinity and temperature over time. Average in-situ pH_TS_ values over the duration of the experiment were 8.027 (±0.005 SD), 7.937 (±0.005) and 7.793 (±0.007) for the 410 µatm (control), 510 µatm and 730 µatm pCO_2_ treatments respectively. Average pCO_2_ levels over the duration of the experiment were 408.6 (±6.2 SD), 512.0 (±7.0) and 730.2 (±14.0) µatm for the 3 treatments. Average aragonite saturation values (Ω_ara_) for the three treatments were 1.35 (±0.02 SD), 1.12 (±0.01), and 0.82 (±0.01).

### Developmental Progression

**Figure 3:**
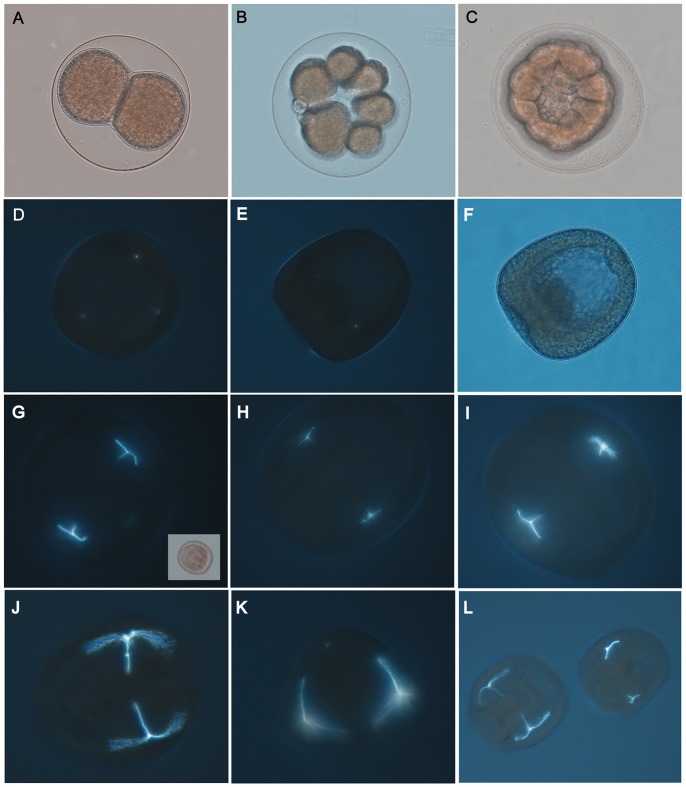
Developmental series of embryo images, including polarized microscopy. Developmental stages and skeletal development of *S. neumayeri* under elevated pCO_2_. A–C) Representative embryos at 2 cell, 16 cell and morula stages reared under ambient (unmanipulated seawater) conditions in a separate culture. D–F) Eight day old early gastrula with vegetal plate indentation where CaCO_3_ spicule nuclei are first visible. Three spicule nuclei are visible under polarized light (D) from the ventral view of the embryo grown in the control treatment. Polarized (E) and brightfield (F) side views of a gastrula grown in 730 µatm pCO_2_ treatment. Two spicule nuclei among the mesenchyme cells are visible from this angle. G–I) Ten day old mid-gastrula embryos (also G-inset) from three treatments: control (G), 510 µatm (H) and 730 µatm (I). Triradiate spicules are similar in shape. J–L) Twelve day old late gastrula stage embryos in three treatments. Large well-developed spicules are visible from the side (J, L) and ventral view (K). Aberrant spicule development is readily distinguishable in some individuals in the 730 µatm treatment (L).

The development of *S.neumayeri* embryos in our culture system followed a developmental schedule slightly accelerated relative to a previously published schedule for cultures grown at McMurdo Station (Table 1); for example, this culture developed to early pluteus 4 days faster. As water temperatures within our culturing system were slightly elevated relative to water temperatures reported in Bosch *et al*. [23], the overall faster rate of development is to be expected; additionally differences between culturing conditions (vessel size and water movement) can result in different developmental rates [23]. Early cleavages, from fertilization to morula stage, (Fig. 3A–C) proceeded normally in all treatments with no obvious morphological or developmental delays between treatments. In addition to the hatching times listed in Table 1, our reported hatching time is intermediate between those observed by Bosch *et al.* [23] for embryos hatched in a seawater table and in a refrigerator (122–110 hrs). As development proceeded, the prolonged transition of mesenchyme blastula to early gastrula provided multiple days to observe changes, such as the gradual formation of the archenteron and the spicule nuclei (Fig. 3D–L). The appearance of the spicule nuclei in the mesenchyme blastula stage (day 8, Fig. 3D–E) was synchronous in all treatments. Early spicule growth appeared similar between treatments (Fig. 3G–I) in the mid-gastrula stage, where the triradiate spicules were extending along all three axes on both sides of the embryo symmetrically.

**Table 1:** Developmental schedule (days post-fertilization) of *S. neumayeri* reared in water from McMurdo Sound – for this study and for data reported by Bosch *et al.* [23].

	This study	Bosch *et al.* [23]: Table 1	
Stage	−0.8 to 0°C	−0.5 to +0.5°C*	−1.8 to −0.9°C
4-cell	0.7		
8-cell	1.0		
Early blastula	2.0	1.7	2.1
Early blastula with cilia	3.2		
Unhatched motile blastula	4.1		
Hatching	4.7	3.7	5.1
Mesenchyme (M) blastula	5.3		
Early gastrula§ with spicule	7.9	8	10
Mid-gastrula	8.8		
Late gastrula	9.3		
Stomatodeal breakthrough	12		
Prism	15	15	16
Early 4-arm pluteus	17	17	21

They have reported the first appearance of stages while our report is the stage of the majority of embryos observed.

* Cultures grown in Santa Cruz, CA, USA.

§ The indentation of the vegetal plate was initiated but the archenteron was not yet formed. See also Fig. 3F.

**Figure 4:**
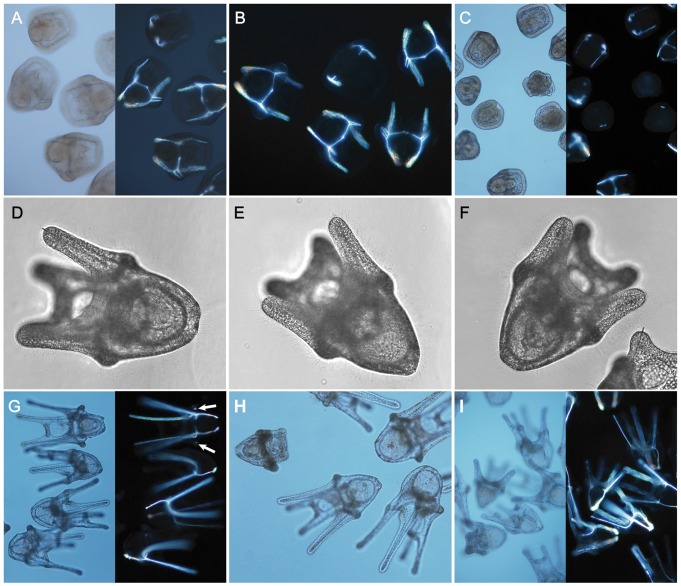
Developmental series of larvae images, including polarized microscopy. A–C) Fifteen day old prism larvae imaged under brightfield and polarized light. Aberrant skeletal and morphological development is found in the 510 µatm (B) and 730 µatm (C) treatments. D–F) Twenty day old 4-arm plutei in three treatments as imaged for morphometric measurements. G–I) Thirty day old plutei. Spicule nuclei for the developing posterodorsal arms are visible in the polarized light image of control treatment larvae (G-arrow). Smaller larvae with short arms are visible in the 510 µatm and 730 µatm treatments (H, I).

However, as development proceeded to the late gastrula stage (day 12), the lengthening of the spicules was not the same between treatments (Fig. 3J–L, see also below). Some aberrant development, both in gross morphology and skeletal elements, was observed in elevated CO_2_ treatments from late gastrula and prism (Fig. 3L, Fig. 4B–C), though these abnormal individuals constituted only a small proportion (see below). Abnormalities included underdeveloped or highly asymmetric skeletal elements and signs of developmental arrest in gross morphology. At sampling on day 20, developmentally arrested larvae were no longer observed in cultures. Gross morphology of plutei appeared normal as canonical 4-arm echinoplutei, across treatments with no signs of increased asymmetry (Fig. 4D–I). The shapes of most skeletons in prisms (Fig. 4A–C) and plutei (Fig. 4D–I) were normal in all treatments even though the arms were visibly shorter in the high pCO_2_ treatments. Spicule nuclei of the posterodorsal arms (Fig. 4G - arrow) were visible in some individuals at all treatment levels but were most apparent in the control.

### Developmental Staging

**Figure 5:**
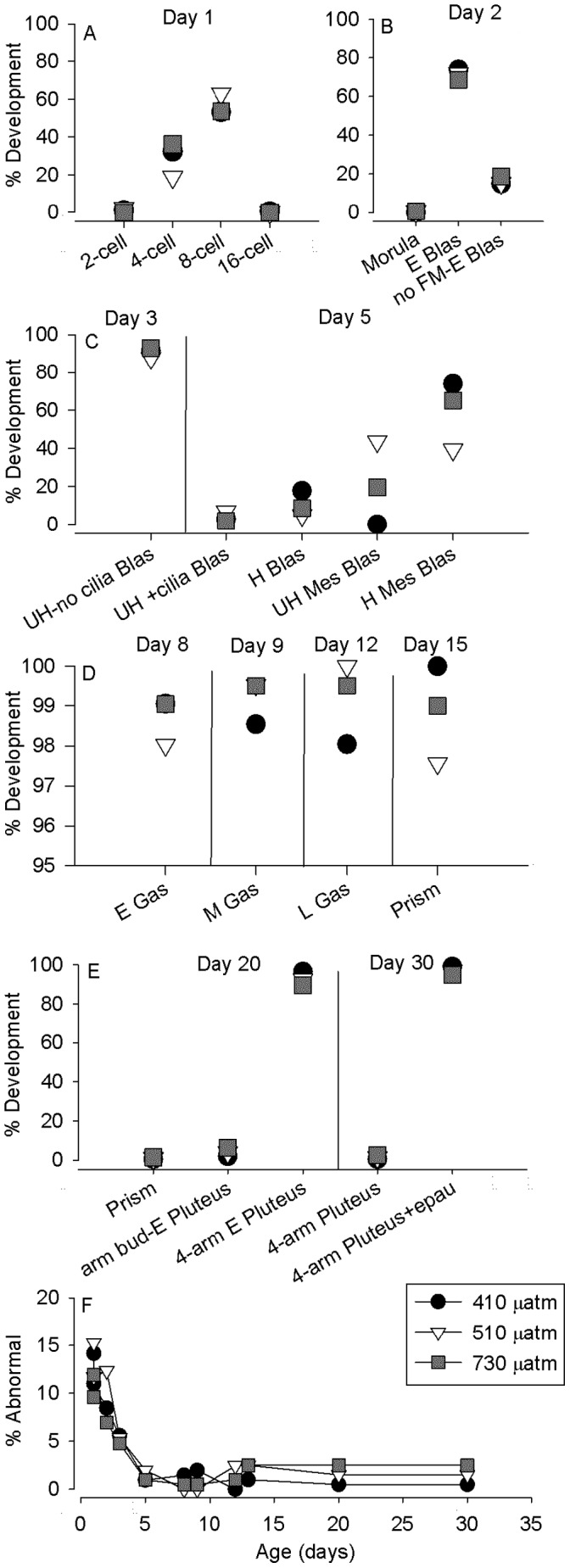
Developmental progression and synchrony at specific times over the duration of the experiment. Samples for each specified day (n>200, each treatment) were scored for developmental stage. A) Relative percentages of normally developed 2–16 cell embryos at 1 day post-fertilization. B) Relative percentages of morulas, and early blastulas (E Blas) at Day 2. Some normally developed embryos were missing a fertilization membrane (no FM-E Blas). C) Day 3 and 5 development. Unhatched (UH) blastulas were observed without cilia (UH-no cilia Blas) on day 3, while ciliated blastulas in various stages were observed at Day 5. Earlier-stage blastulas were unhatched but ciliated (UH+cilia Blas-inset left) or hatched (H Blas). More developed mesenchyme blastulas (Mes Blas) with a distinct vegetal plate were also unhatched and hatched (inset right). D) Gastrulation over days 8–15. Note the change in Y-axis scale. Early gastrula (E Gas) mid-gastrula (M Gas) late gastrula (L Gas) and prism were observed in highly synchronous development. E) Pluteus development at days 20 and 30. Prism and early pluteus (E Plu) were observed at day 20. By day 30 the 4-arms of the plutei are well-developed and some have developed epaulettes (+epau). F) Abnormal development across the experimental period. Percentage of unfertilized and abnormally developed embryos and larvae are shown for the three pCO_2_ treatments.

Fixed larvae were quantitatively staged over development to look for evidence of developmental delay resulting from elevated pCO_2_ (Fig. 5). In general we observed similar (and not significantly delayed) developmental progression at almost every major developmental stage sampled. Embryos at day 1 showed the greatest variation in cell divisions (Fig. 5A), from 2-cell to 16-cell with elevated pCO_2_ treatments developing slightly faster than controls (Fisher’s exact test, p<0.01); at later stages (Fig. 5B–E), stage differences were more subtle and there appeared to be more synchrony. One consistently observed (but rare) abnormality in early embryos was premature loss of the fertilization envelope; this was scored separately at day 2 as “no fertilization membrane early blastula” (no FM-E Blas), as distinct from early blastulas with the fertilization membrane intact (E Blas: Fig. 5B). A small percentage of abnormally developed larvae were observed in all treatments throughout development, but there were no differences between treatments (Fisher’s exact test on proportions of abnormal embryos over time: df = 29, p>0.9) (Fig. 5F).

Unhatched blastulas without cilia (UH-no cilia Blas: Fig. 5C) on day 3 were similar to the stage E Blas on day 2. The hatching of blastulas at day 5 was differentiated somewhat between treatments (Fig. 5C). On day 4 prior to hatching, ciliated, motile blastulas were observed in the culture (Table 1). At day 5, earlier-stage blastulas were unhatched but ciliated (UH+cilia Blas-inset left) or hatched (H Blas). Mesenchyme blastulas with a distinct vegetal plate and animal-vegetal patterning were observed with fertilization membranes (UH Mes Blas) in the 510 and 730 µatm treatments suggesting delayed hatching relative to remaining blastulas (H Mes Blas). Analysis with Fisher’s exact test is highly significant (p<<0.001) for stage differences at day 5.

Gastrula development, from days 8 to 12 was largely synchronous between treatments (Fisher’s exact tests for each sampling day, p>0.05) (Fig. 5D). Indentation at the vegetal pole was the characteristic of early gastrula (E Gas) at day 8, and archenterons halfway extended (mid-gastrula, M Gas) and fully extended (late gastrula, L Gas) were observed in samples from day 9 and 12 respectively. There was a single “unhatched mid-gastrula” at day 9 observed in the 510 µatm treatment [M. Sewell, pers. obs.].

In early plutei at day 20 (Fig. 5E) there was differentiation of external features such as arm buds (arm bud-E Plu) vs. more clearly developed arms (4-arm E Plu). Because larvae were unfed in this experiment, developmental progression was drastically slowed, though arm extension and the appearance of other morphological characters continued. In later plutei at day 30, epaulettes, thickened lobes of ciliated band epithelium on the body that have elongated cilia [23], were observed on the majority of plutei (4-arm pluteus+epau): an indication of continued larval maturation in the absence of food. Differences at prism and pluteus stages were not significant between pCO_2_ treatments (Fisher’s exact tests for each sampling day, p>0.05).

### Morphometrics

**Figure 6:**
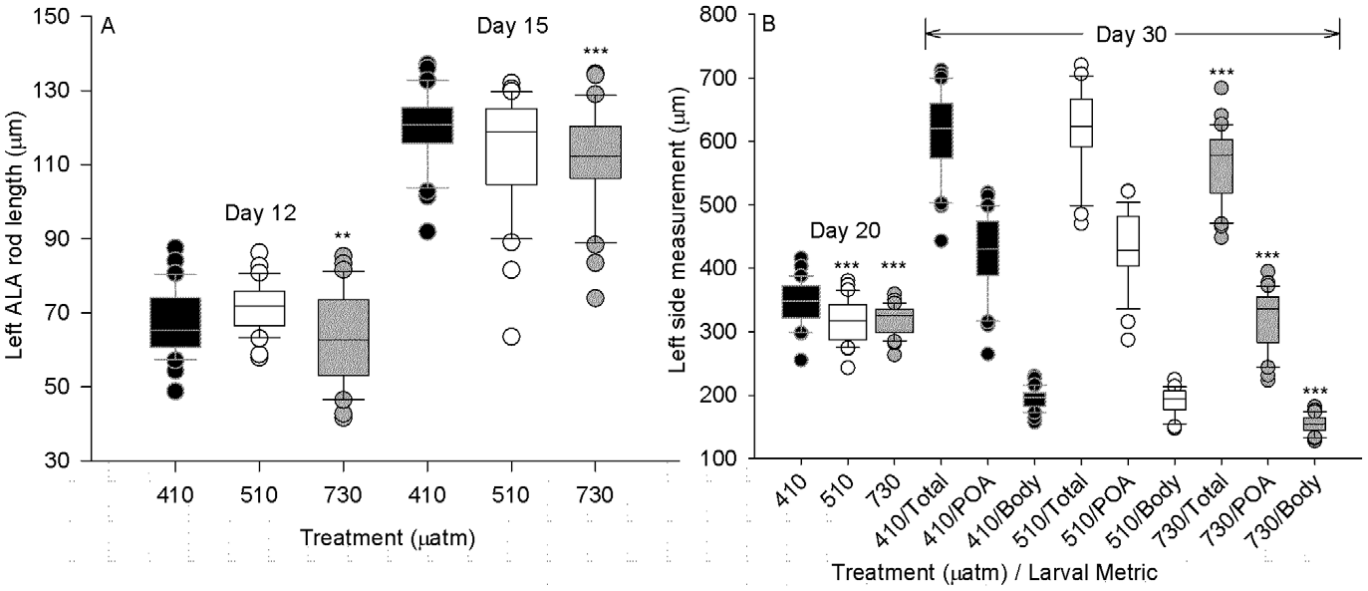
Morphometrics of skeletal elements and larval size from late gastrula to 4-arm pluteus (from day 12–30). Box plots show median, 25th and 75th percentile within the boundaries of the box, 10th and 90th percentiles in the error bars and three lowest and highest outlier values. See Fig. 1 for diagram of measurements. See Tables 1 and 2 for statistics on variances and differences between treatments. A) Anterolateral arm (ALA) rod length (left side) during late gastrulation to prism transition (n = 30). B) Total length in early (day 20) and advanced (day 30) 4-arm plutei. Also shown for day 30 plutei are the postoral arm (POA) rod lengths and body rod lengths (n = 30, except n = 29 for 510 µatm at day 30).

In order to quantify the size differences between treatments, the embryos and larvae were measured on their largest, most readily quantifiable metrics for their developmental stage: the ALA rod for embryos, and TL (and POA and body rods where applicable) for pluteus (see Fig. 1). Because variances were highly unequal between treatments at most sample points, PERMANOVA, a permutation-based ANOVA was implemented due to its insensitivity to both non-normality and heteroscedasticity (Table 2). Dispersion of measurements was significantly heterogeneous in late gastrulae at day 12 (p<0.004) between treatments, but homogeneous at all other stages. Lengths of both skeletal elements and larvae were significantly different between treatments at all stages. At day 12 the 510 µatm treatment was significantly larger than the 730 µatm treatment gastrulae, but not significantly different from the control 410 µatm treatment (Fig. 6A, Table 2). In the prism (day 15) stage, the ALA rods were significantly shorter in the 730 µatm treatment (Fig. 6A, Table 2); the differences between 410 and 730 µatm treatments were significant in every measurement from day 15 onward.

**Table 2:** Permutational homogeneity of dispersion (PERMDlSP2), PERMANOVA and permutational pairwise statistics on morphometrics data.

Age	Measurement	Permutational dispersion(PERMDISP2)	PERMANOVA p	Pairwise tests between treatments
12	Anterolateral arm (ALA) rod length	0.004**	0.005**	730<510 410 = 730 410 = 510
15	ALA rod length	NS	0.037*	730<410 410 = 510 510 = 730
20	Total length	NS	0.001**	730<410 510<410 510 = 730
30	Total length	NS	0.001**	730<410 730<510 410 = 510
30	Postoral (POA) arm rod length	NS	1×10^−4^***	730<410 730<510 410 = 510
30	Body rod length	NS	1×10^−4^***	730<410 730<510 410 = 510

**Figure 7:**
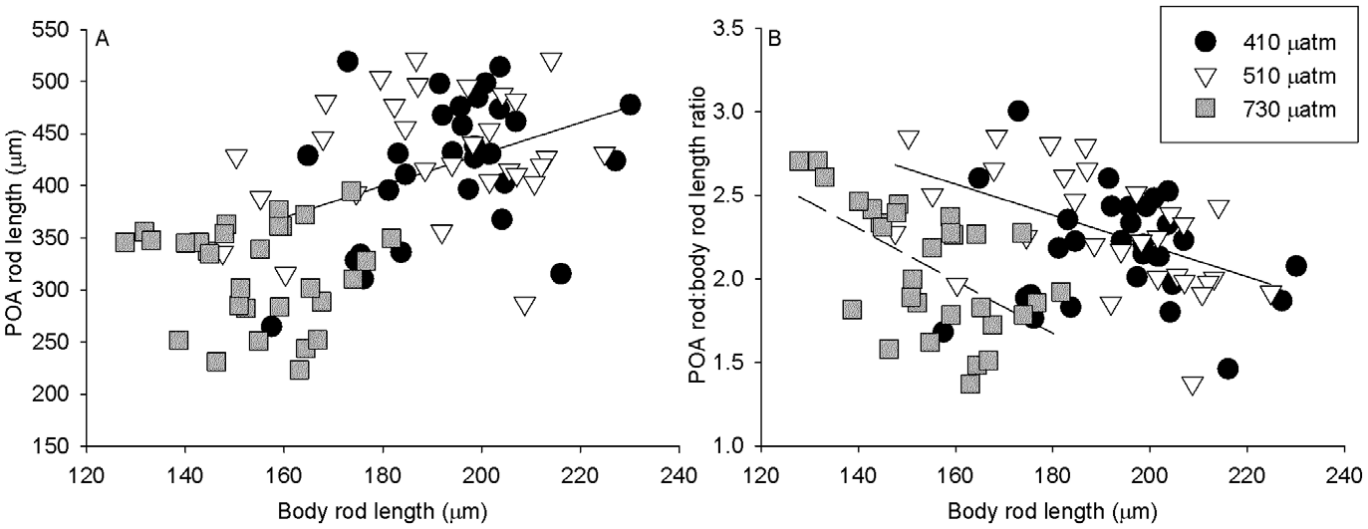
Allometric comparisons of body and arm lengths at day 30. Allometry of individual day 30 plutei considering body rod length as an independent factor. A) The relationship between body rod length and postoral arm (POA) rod length for all assayed individuals in 3 pCO_2_ treatments (n = 30 for 410 and 730 µatm treatments, n = 29 for 510 µatm). POA rod length is correlated significantly to body rod length (r^2^ = 0.14, p<0.04) only in the control (410 µatm) treatment (solid line; regressions not shown for NS correlations). B) Relationship of body rod length to POA rod:body rod length ratio. There was a significant correlation at 510 µatm (solid line, r^2^ = 0.29, p<0.003) and at 730 µatm (dashed line, r^2^ = 0.32, p<0.002). NS correlation for control not shown.

By the pluteus stage, the total lengths of the larvae were consistently smaller in the 730 µatm treatment than in the control 410 µatm treatment (Fig. 6B, Table 2). Only at day 20 were the 510 µatm treatment larvae more similar to 730 µatm than to 410 µatm, while the day 30 measurements show no significant difference between 410 and 510 µatm treatment larvae in lengths. At day 30, these total length differences in the 730 µatm treatment were primarily the result of arm length differences (see also Fig. 7), though both arm and body rod lengths were shorter than both control and 510 µatm larvae. The percent contribution of arm length to total larval length was significantly less at 730 µatm (ANOVA, p<<0.001) than at lower pCO_2_ levels (56.1% vs 68–69%, not shown). Because arm length comprises most of total larval length the two will always be correlated, but body rod length is less plastic [48].

Allometry of advanced 4-arm plutei was performed on length measurement data to examine the possible differential effects of CO_2_ treatments on arm and body size. Individual differences in response between treatments were reflected in different allometric relationships. Allometric analysis on the morphometrics of individual plutei at day 30 (Fig. 7A) shows that there is complete overlap between control 410 and 510 µatm treatments while the 730 µatm treatment is distinct. The differences in variance between treatments in POA and body rod lengths are readily apparent (see also Fig. 6B). Despite the clear overlap in distributions between control and 510 µatm, POA rod length was significantly related to body rod length (r^2^ = 0.14, p<0.04) only in the control 410 µatm treatment; the two measures were not significantly related at elevated pCO_2_ (Fig. 7A). The low r^2^ within all treatments suggests that the ratio of POA to body rod length (POA rod:body rod) is fairly plastic between individuals within a population, even between half-siblings, and this ratio is not significantly different between treatments (ANOVA, p>0.6). Despite the similarities in POA rod:body rod between treatments, only at elevated pCO_2_ was there a significant relationship (510 µatm: r^2^ = 0.29, p<0.003; 730 µatm: r^2^ = 0.32, p<0.002) between POA rod:body rod and body rod length (Fig. 7B).

## Discussion

The near-future potential for year-round calcium carbonate undersaturation in polar oceans indicates that lightly calcified Antarctic invertebrate fauna could be more vulnerable to mildly elevated levels of CO_2_ than organisms in warmer marine ecosystems [3], [48]. In this study, we investigated the effects of elevated pCO_2_ on the larvae of the common benthic echinoderm *Sterechinus neumayeri*. Over the course of development from egg to late 4-arm pluteus, we found (1) early embryological development was normal with the exception of the hatching process, which was slightly delayed, (2) the onset of calcification as determined by the appearance of CaCO_3_ spicule nuclei was on schedule, (3) the lengths of the spicule elements, and the elongation of the spicule nuclei into the larval skeleton, were significantly shorter in the highest CO_2_ treatment 4 days after the initial appearance of the spicule nuclei, (4) finally, without evidence of true developmental delay, larvae were smaller overall under high CO_2_ treatments and arm length, the most plastic morphological aspect of the echinopluteus, exhibited the greatest response to high pCO_2_/low pH/low carbonate conditions.

### Seawater Conditions: in the Field and in the Lab

In order to adequately parameterize the control conditions for our experiment, we sampled seawater from the Cape Evans area to assess present day levels of carbonate chemistry where urchin larvae would be present in the plankton. Current water conditions at McMurdo Sound are already at low Ω_ara_ saturation levels (1.24 at Cape Evans [20]), and Antarctic waters are predicted to become aragonite undersaturated (Ω_ara_ <1) in the very near future on a seasonal basis [4], [17], and completely undersaturated a few decades thereafter [1], [49]. Thus for our experiments we targeted ∼400 µatm/Ω_ara_ = 1.24 for ambient levels, and Ω_ara_≈1 and Ω_ara_<1 for our elevated pCO_2_ future climate treatments; the discrepancy between culture temperatures and environmental temperatures prevented us from more precisely matching our target values.

We then tested the effects of elevated CO_2_ levels on early development of *S. neumayeri* in seawater from McMurdo Sound, and found that embryos and larvae were developmentally robust to the experimental conditions. Rates of survival, while not directly measured, were high in all CO_2_ treatments (410, 510 and 730 µatm) over the duration of the experiment. Previous research on larvae of *S. neumayeri* reported survival differences only at very severely lowered pH levels (pH_NBS_ <6.5) [29]. Similarly, deleterious effects during embryonic development were reported only at low pH (pH_NBS_ ≤7.3, which would correspond to pCO_2_>2880 µatm: [13], and pH_TS_ = 7.5: [30]).

### Developmental Progression

In order to address the potential confounding of decreased growth and delayed development, we monitored developmental progression of larvae in order to ascertain whether developmental delays would result from elevated pCO_2_ conditions. Developmental scheduling in all CO_2_ treatments was similar to previously reported data for this species (Table 1). Because of the warmer temperatures indoors in the Crary Lab (Fig. 2C), cultures could not be consistently maintained at the true environmental temperature of −1.9°C, and as a result, the developmental rate of our embryos was accelerated relative to the rates reported by Bosch *et al.* [23], particularly in comparison to the hatching time reported for embryos cultured *in situ* in McMurdo Sound (5.8 days: [23]).

Early embryos were indistinguishable in appearance between all CO_2_ treatment groups, demonstrating a lack of effect on basic cell-division and morphogenesis. One study reported deleterious CO_2_ effects on early cell divisions only at sub-optimal sperm concentrations [13]; other evidence demonstrates an overall negative effect of elevated pCO_2_ but that responses can be variable between male-female pairs [50]. Elevated temperature has deleterious effects on early cleavages [51], but not with synergistic effects of low pH [30]. Another study on the possible mechanism of delays at first cleavage in *Strongylocentrotus purpuratus* under highly elevated CO_2_/low pH (pH 7.0/4000 ppm pCO_2_) demonstrated no deleterious effects on cell cycle checkpoints [52]. Developmental progression from early blastula to mesenchyme blastula in *S. neumayeri* was similar between treatments but hatching rates were not. Differences in development were most obvious at day 5, the hatching blastula stage (Fig. 5C). There have not been previous reports regarding the effects of CO_2_ on hatching, but research on enzymatic activity of purified hatching proteases from temperate sea urchin species suggest that pH 8 is optimum for maximal activity *in vitro* [53], [54]. Though hatching was delayed, the embryos developed on schedule within the envelope, which suggests a decoupling of the hatching process from the other more critical developmental pathways; echinoid embryonic development can proceed normally in the absence of a fertilization envelope in culture and the removal of the envelope is required for techniques such as blastomere separation [55], [56]. Delayed hatching with developmental progression in echinoids has now been reported as a response to salinity stress [57], so our observation of delayed hatching is perhaps indicative of an environmental stress response.

Developmental progress was synchronous from gastrulation onward with non-significant differences in developmental staging between treatments over the course of development to pluteus. Gastrulation is a critical developmental regulation point [58] at which major gene expression changes can also result in mortality due to genetic load in high fecundity invertebrates [59], [60]. Low percentage of gastrulation abnormalities at mildly elevated pCO_2_ treatments is consistent with previous reports in *S. neumayeri* (for pH_NBS_ ≥7.7: [13]), and in *Strongylocentrotus purpuratus* [61]. Gastrulae and larvae with skeletal anomalies (of the varieties seen in Fig. 3L, Fig. 4B and C) were observed in all treatments (Fig. 5F). Studies in *Strongylocentrotus purpuratus* have failed to detect delays in the spicule nuclei appearance prior to skeleton elongation under elevated pCO_2_ [61], and control over spiculogenesis and the initial precipitation of these CaCO_3_ nuclei is distinct from spicule elongation processes in the primary mesenchyme cells [62], [63].

### Morphometric Differences

The significantly smaller larvae observed in the 730 µatm treatment are consistent with observations of less growth under elevated CO_2_/low pH in the larvae many other species of sea urchin when tested under future climate scenarios [35], [37], [39], [64]. However while the pCO_2_ treatment level (730 µatm) which has effected this difference (∼8% at all 4 measured stages) is much lower than has been used with experiments on temperate larvae (compare with effects at 1000 µatm in *Strongylocentrotus purpuratus:* [65]), the Ω_ara_ difference tested on the polar species is smaller and going from saturation (Ω_ara_ = 1.35) to undersaturation (Ω_ara_ = 0.82). The Antarctic species appears to respond with greater sensitivity than temperate species to smaller changes in pCO_2_/pH/Ω because it currently lives in conditions that are already difficult for calcification. The use of Ω_ara_ rather than Ω_cal_ for comparison is appropriate due to the Mg^+2^ content of calcite deposited by echinoderms [5], [66].

The size difference between treatments in ALA rods at late gastrulation shows that the differences are detectable early in the skeleton development process, even though there was no substantial delay in the first appearance of the initial spicule nuclei between treatments. Differences in size of the POA rod at day 30 show that the differences in total length are largely attributable to POA length differences. This finding is unsurprising given that arm length is the most plastic attribute of echinopluteus larvae, and can be readily modified in response to food cues [67], [68], [69], [70] and pharmacological manipulation [71]. Recent studies [72], [73] have asserted that developmental delay is solely responsible for the smaller size of larvae under elevated CO_2_. Because the plasticity of the feeding arms in echinoid larvae is controlled by food and hormonal cues [70], [71], [74] arm length may not represent a reliable “fixed” metric from which to determine relative age.

The skeletal elements of larvae are significantly and consistently smaller in the 730 µatm treatment at days 15 and 30. While there were overall significant length differences between treatments at day 12, the significant dispersion (as tested by PERMDISP) appears to be between the two elevated pCO_2_ treatments, and greater size variance at elevated pCO_2_ has been observed in other sea urchin larvae [75]. If a delay and/or slower growth had occurred to make larvae at high CO_2_ smaller, this difference may have occurred subsequent to the earliest growth period after day 12. Body rod length does not appear to increase in this species after the attainment of the pluteus in the absence of food [P. Yu, unpub.], as reported in some other species of echinoid [47], [74]. The smaller body rod size of advanced 4-armed larvae in *S. neumayeri* suggests that smaller body size under high pCO_2_ is not a growth intermediate, but could either be fixed at attainment of the pluteus stage at a smaller size, or even be the result of shrinkage during food deprivation. The allometric changes in response to elevated pCO_2_ (Fig. 7A: loss of correlation between POA rod and body rod lengths and Fig. 7B: increasing response of the POA: body rod ratio to body rod length) is also suggestive of true size changes imposed by elevated pCO_2_ as delayed growth would not result in a relaxation of allometric relationships. Larval growth is regulated by cell number, not cell size [76], so future measurements of cell number in this species may clarify some of the observed differences. The simultaneous interaction of growth and development in continuously growing invertebrate larvae is still poorly understood, as compared with the amount of knowledge in arthropods regarding size-age relationships [77].

### Physiological Implications of Size Differences

Due to the simultaneous interaction of growth and development, the effect of slower growth vs. age-delay cannot be readily decoupled developmentally or mechanistically without additional markers of developmental progression in continuously growing larvae [78]. Interpretations of a developmental delay mechanism based on decreased metabolism are inconclusive, as a previous study on metabolic rates in larvae of *S. neumayeri* suggested a decreased metabolic rate under elevated pCO_2_ [79] while in temperate species, size-specific metabolic rates were unchanged [73] or increased [72]. There was no evidence for developmental delay from developmental staging [80] or rates of lipid catabolism [81] under elevated pCO_2_ in *Strongylocentrotus purpuratus* during early development. Gene expression patterns under elevated CO_2_ during early development showed no differences that would indicate developmental delay [82], while other studies have interpreted their results as indicative of developmental delay [83]. The synchrony of overall development observed in our cultures between treatments (Fig. 5) provides strong evidence for a lack of developmental delay and for attenuation in size due to lower growth.

Arm length is important for maintaining buoyancy during swimming and for food capture in echinoplutei. Maintaining longer arms involves growth trade-offs [84], and longer arms correlate with ciliated band length and feeding capacity [84], and organismal lipid content [71], [81]. Though elevated CO_2_ has been repeatedly shown to result in shorter arm lengths, data from Stumpp *et al*. [72] show that size-specific feeding rates are not affected by pH treatment. While we elected to not feed the larvae in this study, the early development of these larvae in their environment during the late austral spring is under nutrient-poor conditions [41]; thus our experimental conditions represent a temporary natural stress for the larvae. Studies on later, more advanced larval development would need to incorporate naturalistic feeding treatments to better ascertain the downstream developmental effects of smaller arm and body size. Studies on juvenile corals have shown that feeding can mitigate some of the effects of low pH on growth and calcification (reviewed in [85]), but the correlation of arm length to feeding in echinoid larvae means that shorter arms from high pCO_2_ will inhibit food intake.

Slower growth and smaller size could lead to longer pelagic larval duration, which for *S. neumayeri* is almost 4 months under optimal conditions [23]. Spawning of *S. neumayeri* is timed slightly in advance of seasonal availability of phytoplankton resources [86], and phenological mismatch during development could potentially occur with climate change. Longer pelagic larval duration can also result in increased pelagic predation risk [87], [88]. Recruitment of *S. neumayeri* is estimated to be sporadic and low, suggesting most annual gonadal production is already consumed by predators [89].

The Ross Sea currently faces a number of environmental challenges. Estimates of future undersaturation are only a few decades away by the most recent model estimates [4]. A slight increase in pCO_2_ (+100 µatm above present day levels) did not consistently decrease size in this species over most of early larval development. Notably, an elevation of pCO_2_ by about twice current atmospheric levels (projected to occur by the year 2050: [49]) resulted in significantly smaller skeletons and bodies, but did not noticeably change rates of early development in a slow-growing species. Future warming will accelerate development times, but may then result in undersized larvae with simultaneously elevated pCO_2_ conditions. Based on demographic surveys, the lifespan of *S. neumayeri* is estimated to be roughly 40 years in the McMurdo Sound region [89], making the settlers of the present day the highest fecundity reproductive classes of these future acidified conditions. Currently in parts of the Antarctic Peninsula, predation on adult urchins by invasive crabs present a more immediate threat to this species [8], [9]. Thus there will be a multitude of simultaneous challenges to the future persistence of this species and other shelled invertebrates of Antarctica.
